# Mental Health Status, Life Satisfaction, and Mood State of Elite Athletes During the COVID-19 Pandemic: A Follow-Up Study in the Phases of Home Confinement, Reopening, and Semi-Lockdown Condition

**DOI:** 10.3389/fpsyg.2021.630414

**Published:** 2021-06-11

**Authors:** Amir Hossien Mehrsafar, Ali Moghadam Zadeh, Parisa Gazerani, Jose Carlos Jaenes Sanchez, Mehri Nejat, Mastaneh Rajabian Tabesh, Maryam Abolhasani

**Affiliations:** ^1^Department of Sport Psychology, Faculty of Sports Sciences, University of Tehran, Tehran, Iran; ^2^Department of Psychology, Faculty of Psychology and Education, University of Tehran, Tehran, Iran; ^3^Department of Health Science and Technology, School of Medicine, Aalborg University, Aalborg, Denmark; ^4^Department of Life Sciences and Health, Faculty of Health Sciences, Oslo Metropolitan University, Oslo, Norway; ^5^Department of Social Anthropology, Basic Psychology & Health, Universidad Pablo de Olavide, Seville, Spain; ^6^Andalusian Center of Sport Medicine, Seville, Spain; ^7^Nejat Psychiatric and Sexual Disorders Center, Tehran, Iran; ^8^Sports Medicine Department, Sina Hospital, Tehran University of Medical Science, Tehran, Iran

**Keywords:** mental health, mood states, life satisfaction, COVID-19, elite athletes, sport

## Abstract

Scientific reports notified that the pandemic caused by the Coronavirus disease 2019 (COVID-19) has raised an unprecedented mental health emergency worldwide. Abrupt changes in daily routine, environmental constraints, adopted home confinement measures, and uncertainty about a date for returning to usual activities can potentially affect mental health and sports activities in athletes. Hence, we designed a cross-sectional study with a within-subjects design to investigate the impact of the pandemic on mental health, mood states, and life satisfaction of elite athletes. During the three phases of home confinement (April 14–24, *n* = 525), reopening (May 9–19, *n* = 464), and current semi-lockdown (July 20–31, *n* = 428), elite athletes voluntarily responded to an online survey. The self-report questionnaire was prepared to collect demographic and epidemiological variables of interest and the COVID-19-related information. All participants also completed the Profile of Mood State (POMS), General Health Questionnaire-28 (GHQ-28), and Satisfaction with Life Scale (SWLS). The main result is that the training rate, mental health, life satisfaction, and positive mood have decreased during the home confinement period as compared with the reopening and semi-lockdown phases. However, the need for psychosocial services has increased during the pandemic period. The present study provides the first preliminary evidence that home confinement conditions during the COVID-19 pandemic might have negatively influenced elite athlete’s mood state, mental health, and life satisfaction, as well as training rates. Monitoring the psychological parameters of elite athletes and developing strategies to improve their mental health during the COVID-19 pandemic should be on the agenda. Next studies, therefore, seem reasonable to focus on active interventions for athletes during the ongoing COVID-19 pandemic.

## Introduction

Coronavirus disease 2019 (COVID-19) is an emerging infectious disease caused by newly discovered Severe Acute Respiratory Syndrome Coronavirus 2 (SARS-CoV-2). It was firstly reported in Wuhan, Hubei province (World Health Organization, 2020b), causing an unprecedented pandemic, forcing governments to impose an almost global quarantine (Spinelli and Pellino, 2020). The infection has spread rapidly around the world and COVID-19 has caused many severe and fatal medical cases (Rothan and Byrareddy, 2020). The escalating global morbidity and mortality of COVID-19 have raised significant public health concerns. At present, the focus of governments and the World Health Organization (WHO) is on controlling and mitigating the impact of this pandemic by identifying, testing, and treating infected people, and developing drugs, vaccines, and treatment protocols (Salathé et al., 2020). However, despite such efforts to defeat this pandemic, the directions where the pandemic will take in the coming days unknown. The global community is concerned about COVID-19 and its long-term consequences, such as the economy, industries, global market, human health, and health care. The COVID-19 outbreak also negatively influences various sports activities. In this regard, the 2020 Olympics and many national and international sport events/competitions have been postponed or canceled, and many organized training and team practices were limited. Consequently, many athletes faced tight restrictions to continue their regular training or activities. Health authorities recommend these limitations due to the nature of many team-based activities and crowd attendance that may facilitate a rapid spread of COVID-19, resulting in additional pressure on the health care system (World Health Organization, 2020a). In this regard, big sports events that resumed during the midst of the COVID-19 outbreak are now being referred to as huge “biological bombs” due to the spreading of the virus during these events (Gilat and Cole, 2020).

The positive COVID-19 tests in world-class athletes at premier leagues presented that no one is potentially safe (Corsini et al., 2020). Moreover, according to media, COVID-19 has also been fatal to athletes. Many athletes are also required to train under isolation or home confinement conditions. Their daily routines such as doing primary outdoor activities, going to professional clubs, and utilizing tools counteract the current situation of home confinement and restrictions (Hargreaves et al., 2021). Reports are presented that numerous athletes have been able to overcome challenges to continue training and daily practice by other means and alternatives and adapted to necessary changes during the pandemic. On the other hand, mental health professionals have suggested that exercise could be a way to improve mental well-being during this pandemic (Kar et al., 2020). For instance, home-based physical activity programs may contribute to reducing the symptoms of depression and anxiety (Iannaccone et al., 2020). This might reflect a positive view; however, exercise at an ordinary level might have a different impact compared with those requirements for athletes, in particular, those in preparation for athletic competition. There are still several potential issues that must be considered for the negative impact of the pandemic on athletes (Jiménez-Pavón et al., 2020).

WHO has also expressed its concern over the pandemic’s mental health and psycho-social consequences (World Health Organization, 2020c). It speculates that new measures such as self-isolation and quarantine have affected usual activities, routines, and livelihoods of people that may lead to both physical and mental challenges for athletes (Mehrsafar et al., 2020), including an increase in fear of COVID-19 (Alsalhe et al., 2020), rumination (Satici et al., 2020), anxiety and depression (Gouttebarge et al., 2020), gambling problems (Håkansson et al., 2020), sleep and eating disorders (Pillay et al., 2020), psychological inflexibility (Fuentes-García et al., 2020), obsessive–compulsive disorder (Edwards and Thornton, 2020), family conflicts (Mehrsafar et al., 2020), concerns about not getting back to previous fitness (Clemente-Suárez et al., 2020; Mon-López et al., 2020b), sedentary lifestyle and negative habits (León-Zarceño et al., 2021), low mood (Ammer et al., 2020a), harmful consumption of alcohol and drugs (Clay and Parker, 2020), and self-harm attempts or suicidal behavior (Fegert et al., 2020). Of note, several coaches and specifically sports psychologists and even psychiatrists reported that some psychological disorders have been diagnosed among athletes, and those can pose a negative effect on their life (Ammer et al., 2020b; Schinke et al., 2020). A recent narrative review indicated that the COVID-19 pandemic has created several new stressors for elite athletes and that elite athletes suffer from many mental health symptoms and disorders at rates equivalent to or exceeding those in the general population (Reardon et al., 2020). Besides, researchers state that athletes need to receive psychological counseling also in addition to careful screening and monitoring of athletes during the COVID-19 pandemic (Reardon et al., 2020; Schinke et al., 2020).

It is noteworthy that after the quarantine, the governments decided to reopen the community and, consequently, sports with some recommendations (Jorstad and van den Aardweg, 2020; Ludvigsen and Hayton, 2020). However, a previous study on French athletes has shown higher scores of anxiety upon returning to the competition after lockdown by COVID-19, and also competition is considered a new stressor for elite athletes (Ruffault et al., 2020). In this regard, sports medicine specialists and other managers designed plans and strategies for the participation in sport and return to training and competitions (Mohr et al., 2020; Quchan and Kordi, 2020). However, after reopening, governments reported that the number of COVID-19 patients increased rapidly. There are also reports that some elite athletes have contracted the COVID-19 since reopening. Following the increase in the number of patients and virus mutations, some governments decided to pose semi-lockdown conditions and sports activities are somewhat restricted and many sports clubs are closed again. However, researchers showed that after 7 weeks of reopening of sport, a positive psychological adaptation occurred for the athletes similar to the pre-COVID-19 pandemic (Rubio et al., 2020).

Elite athletes as a target group might need specific help for their psychological needs, including maintenance of mental health during outbreaks to minimize the psychosocial toll. An accurate understanding of the psychological impact accompanying the COVID-19 lockdown, reopening, and semi-lockdown is necessary to plan for a crisis-oriented base research and intervention to foster a healthy lifestyle and physical and mental well-being in athletes (Zhang et al., 2020). Consistent with this standpoint, and to address the call by the scientific and sports community to identify challenges and plans to reduce mental health consequences of the COVID-19 pandemic in athletes, we performed an online survey to determine changes in mental health status, mood state, and adaptive behaviors of elite athletes during the COVID-19 pandemic in home confinement, reopening, and semi-lockdown phases. To the best of our knowledge, no study has examined the status of mental health factors during the reopening and semi-lockdown periods.

## Materials and Methods

### Study Design and Participants

The first phase (home confinement) of the study was conducted through an online survey between April 14 and April 24, 2020. This time frame was chosen to assess participants’ responses during an early phase of the COVID-19 outbreak, following the Iranian Government declaration of lockdown and the WHO announcement of the COVID-19 as a pandemic (March 11, 2020). Moreover, at this time, all sports facilities and clubs were closed and all sports events were canceled. The second phase (reopening condition) of this study was performed between May 09 and May 19, 2020. In this phase, athletes were able to continue sports training with some restrictions and more sports clubs opened. Finally, the third phase (semi-lockdown) of our study was launched between July 20 and July 31, 2020. During this period, the Iranian Government announced the semi-lockdown condition for the community and more sports clubs were closed, and the daily routine of elite athletes was difficult and limited. It should be noted that the second and third phases of this study were performed similarly to the first phase procedure.

Efforts were made to recruit participants from all regions in the country, affected by the pandemic to different extents, to obtain a representative population. Eligible participants were in the age range of 18–45 years, were competing in the super league, national, and international levels (Lorenz et al., 2013); had lived in Iran, and were fluent in both written and spoken Persian. Exclusion criteria were as follows: non-elite athletes, non-Persian language speakers, current hospitalization, and a history of mental disorder. All participants completed the questionnaire online via the Cofepardazesh platform. A link was distributed via a range of methods: invitation via e-mails, shared in faculties and organizations’ official pages, and other social media platforms such as Telegram^TM^, Instagram^TM^, and WhatsApp^TM^. It is necessary to mention that athletes and some research and sports organizations were also involved in the dissemination plans of our research through the promotion of the survey in their networks.

### Ethics Statement

The study was approved by the Ethics Committee of the Tehran University of Medical Sciences (number: 1399-335) and was conducted according to the Declaration of Helsinki. The participant could withdraw from the survey at any moment without providing any justification. We also provided scores and status reports for anyone who wished. The survey was anonymous, and data confidentiality was assured according to the privacy policy. This web-based questionnaire was completely voluntary and non-commercial.

### Data Collection

The survey included an introductory page describing the background and the aims of the survey and the ethics information for participants. A dedicated, self-report questionnaire was set up to collect demographic and epidemiological variables of interest (age, gender, marital status, life form, educational level, sport type, and competitive levels), information on lockdown conditions (exercise before COVID-19^h/w^, exercise during COVID-19^h/w^, and coaches follow-up for plans and fitness and COVID-19-related information (infection, acceptance of quarantine rules and COVID-19 instructions, economic damage, and need for psychosocial services). All participants completed five standardized Persian versions of these questionnaires including General Health Questionnaire-28 (GHQ-28), Profile of Mood State Short Form (POMS-SF), and Satisfaction with Life Scale (SWLS).

To ensure the quality of the survey, we set the response range of some items (some items needed to be answered in reverse) and encouraged participants to answer carefully through questionnaire explanations. Moreover, questionnaires that were completed less than 1 min or more than 20 min would be excluded from the analysis.

### Psychometric Assessments

#### General Health Questionnaire-28 (GHQ-28)

General Health Questionnaire-28 (GHQ-28) has been developed by Goldberg (1978) and Sterling (2011). This questionnaire has 28 items and four subscales including somatic symptoms (items 1–7), anxiety (items 8–14), social dysfunction (items 15–21), and severe depression (items 22–28). This scale is a four-point Likert scale from not at all (score 0), no more than usual (score 1), rather more than usual (score 2), to much more than usual (score 3). The total possible score on the GHQ 28 ranges from 0 to 84 and the high score in this questionnaire indicates a greater disorder in the mental health of individuals. The reliability of the Persian form of the GHQ-28 using Cronbach’s alpha coefficient was acceptable in the range of 0.73 to 0.89. Moreover, four factors have been extracted by using factor analysis and the principal component analysis, and the confirmatory factor analysis confirmed four subscales with 28 items (Noorbala and Mohammad, 2009).

#### Satisfaction With Life Scale

The SWLS was designed as a measure of individuals’ global judgment of their life (Diener et al., 1985). The SWLS consists of five statements, scored on a scale ranging from 1 (completely disagree) to 7 (completely agree). The total score is the sum of the item scores (range, 5–35). The validity and reliability of SWLS have been confirmed in the Iranian population (Maroufizadeh et al., 2016). In more detail, the Cronbach’s alpha reliability of the scale was 0.90, and also confirmatory factor analysis confirmed a one-factor model with good fit statistical indexes.

#### Profile of Mood State Short Form

The POMS-SF is a reliable and valid measure of subjective mood states that has been used extensively in a wide variety of studies (Chen et al., 2002). This questionnaire has 30 items and six subscales including tension, depression, anger, vigor, fatigue, and confusion. Participants respond on a five-point, Likert-type scale ranging from 0 (not at all) to 4 (extremely). Each of these six dimensions is defined by five adjectives. The reliability of the Persian of POMS-SF using Cronbach’s alpha coefficient was acceptable in the range of 0.74 to 0.88. Besides, the confirmatory factor analysis has confirmed a six-factor model as a good fit with strong statistical evidence (Farrokhi et al., 2012).

### Statistical Analysis

The data were analyzed using the Statistical Package for the Social Sciences (SPSS, version 22.0, Chicago, IL, United States). Data were checked for missing, outliers, and normal distribution. The Kolmogorov–Smirnov normality test showed that most variables were not normally distributed. Values were computed and reported for illustrating purposes as mean ± SD (standard deviation) and frequency (*n*) and percentage (%).

To test the effect of COVID-19 pandemic condition on the self-reported measure (training during COVID-19 pandemic), a repeated measure analysis of variance (ANOVA) with a within-subject factor was conducted for the three conditions (home confinement, reopening, and semi-lockdown). Moreover, we applied Bonferroni correction for multiple comparisons, when the ANOVA outcomes were significant. For those variables without normal distribution and ordinal variables, we utilized Friedman tests for comparison of the three conditions (home confinement, reopening, and semi-lockdown) and subsequently, Wilcoxon Signed Ranks tests as *post hoc* comparisons. The nominal variable (COVID-19 infection) was analyzed using Cochran’s *Q* test for comparison of three conditions and McNemar Tests were utilized as *post hoc* comparisons. Furthermore, Kruskal–Wallis and Mann–Whitney *U* tests were utilized for the comparison of psychological variables in gender, age categories, types of sport, and competitive levels in each phase of this study. Pearson correlation tests were used to assess possible relationships in the difference scores (Δ) of three phases of study between the assessed psychological parameters (mental health, life satisfaction, and mood states). Statistical significance was evaluated as *p* < 0.05 for all tests.

## Results

For the first phase (home confinement condition), 525 (100%) valid responses were received. Our sample decreased by 61 (11.6%) people in the second phase (*n* = 464). In the third phase (semi-lockdown condition), 36 (*n* = 97, 18.5%) people did not participate compared with the second phase (reopening condition) (*n* = 428). Descriptive statistics presenting the demographic information of the participants, including age, gender distributions, educational level, marital status, life form, sports type, and competitive levels, are summarized in Table 1.

**TABLE 1:** Participants’ demographic characteristics.

**Variables/Phase**	**Home confinement condition**		**Reopening condition**		**Semi lockdown condition**	
	**(*N* = 525)**		**(*N* = 464)**		**(*N* = 428)**	
	**Mean or *n***	**±*SD* or valid %**	**Mean or *n***	**±*SD* or valid %**	**Mean or *n***	**±*SD* or valid %**
Age (years)	27.85	±9.09	27.53	±8.74	27.43	8.56
18–25 (years)	256	48.8%	228	49.1%	210	49.1%
26–30 (years)	106	20.2%	99	21.3%	92	21.5%
31–35 (years)	69	13.1%	59	12.7%	55	12.9%
36–40 (years)	50	9.5%	42	9.1%	38	8.9%
41–45 (years)	44	8.4%	36	7.8%	33	7.7%
**Gender**						
Male	205	39.0%	183	39.4%	169	39.5%
Female	320	61.0%	281	60.6%	259	60.5%
**Marital status**						
Single	367	69.9%	326	70.3%	300	70.1%
Married	140	26.7%	121	26.1%	111	25.9%
Divorced, Widow/widower	18	3.4%	17	3.7%	17	4.0%
**Life form**						
Alone	39	7.4%	33	7.1%	28	6.5%
With family	486	92.6%	431	92.9%	400	93.5%
**Education level**						
Less than high school and High school graduate	120	23.0%	117	25.2%	107	25.0%
Bachelor’s degree	231	44.3%	205	44.2%	191	44.6%
Master’s degree	128	24.6%	106	22.8%	97	22.7%
Doctoral degree	42	8.1%	36	7.8%	33	7.7%
**Sport type**						
Individual sports	268	51.0%	228	49.1%	206	48.1%
Team sports	257	49.0%	236	50.9%	222	51.9%
**Competitive levels**						
International	164	31.7%	151	32.5%	138	32.2’%
National	214	41.4%	194	41.8%	186	43.5%
Super league	139	26.9%	119	25.6%	104	24.3%

The mean age of participants was 27.85 ± 9.09 years at the home confinement condition, 27.53 ± 8.74 years at the reopening condition, and 27.43 ± 8.56 years at the semi-lockdown condition. Of the total sample, 205 (39%) were men and 320 (61%) were women at the first phase of this study. In the second phase, 39.4% of the sample were men and 60.6% were women. In the third phase, we observed that 169 (39.5%) of the sample were men and 259 (60.5%) were women; 69.9% of our sample were single and 26.7% were married and 3.4% were divorced or widow/widower at the first phase. In the second phase, 70.3% were single, 26.1% were married, and 3.7% were divorced or widow/widower. At the final phase, 70.1% were single, 26.9% were married, and 4% were divorced or widow/widower. Only 7.4% of the elite athletes lived alone and 92.6% were with family. The same results were found at the second and third phases. The elite athletes were active in individual (home confinement condition: 51%, *n* = 268; reopening condition: 49.1%, *n* = 228; semi-lockdown condition: 48.1%, *n* = 206) and team (home confinement condition: 49%, *n* = 257; reopening condition: 50.9%, *n* = 236; semi-lockdown condition: 51.9%, *n* = 222) sports. Most respondents competed at the national level (home confinement condition: 41.4%; reopening condition: 41.8%; semi-lockdown condition: 43.5%), followed by at the international level (home confinement condition: 31.7%; reopening condition: 32.5%; semi-lockdown condition: 32.2%) and super league (home confinement condition: 26.9%; reopening condition: 25.6%; semi-lockdown condition: 24.3%).

Table 2 describes the personal situation of the respondents about economic damage by COVID-19, acceptance of the rules of quarantine, and health recipes of COVID-19, COVID-19 infection, training during the COVID-19 pandemic (at home and clubs), coaches follow-up, and need for psychosocial services during the pandemic. Moreover, the participants reported their training rate before the COVID-19 pandemic (hours/week: 11.02 ± 8.58).

**TABLE 2:** COVID-related characteristics influencing the participants during the three phases of study.

**Variables/phase**	**Home confinement condition**		**Reopening condition**		**Semi lockdown condition**		***F*/χ^2^/Cochran’s Q**	***p***
	**(*N* = 525)**		**(*N* = 464)**		**(*N* = 428)**			
	**Mean or *n***	**±*SD* or valid %**	**Mean or *n***	**±*SD* or valid %**	**Mean or *n***	**±*SD* or valid %**		
**Economic damage by COVID-19**								
Completely	54	10.3%	224	52.3%	261	56.3%	217.11	0.001
To some extent	252	48.0%	154	36.0%	153	33.0%		
Not at all	219	41.7%	50	11.7%	50	10.8%		
**Acceptance of the rules of quarantine and COVID-19 instructions**								
Completely	243	46.3%	252	54.3%	257	60.0%	27.20	0.001
Most of the time	168	32.0%	139	30.0%	127	29.7%		
Rarely	79	15.0%	53	11.4%	34	7.9%		
Not at all	35	6.7%	20	4.3%	10	2.3%		
**COVID-19 infection**								
No	519	98.9%	454	97.8%	422	98.6%	0.33	0.846
Yes	6	1.1%	10	2.2%	6	1.4%		
**Training during COVID-19 pandemic (at home and clubs)^h/w^**	1.34	±1.14	1.65	±1.20	1.51	±1.07	8.18	0.001
**Coaches follow-up**								
Completely	128	24.4%	116	25.0%	94	22.0%	1.64	0.438
Most of the time	138	26.3%	132	28.4%	122	28.5%		
Rarely	136	25.9%	103	22.2%	99	23.1%		
Not at all	123	23.4%	113	24.4%	113	26.4%		
**Need for psychosocial services**								
Completely	34	6.5%	47	10.1%	69	16.1%	17.15	0.001
To some extent	312	59.4%	267	57.5%	248	57.9%		
Not at all	179	34.1%	150	32.3%	111	25.9%		

As shown in Table 2, the change of economic damage by COVID-19 during the three conditions was significant [χ^2^(2) = 217.11, *p* = 0.001]. Furthermore, Wilcoxon Signed Ranks tests as *post hoc* comparison showed that economic damage by COVID-19 was significantly different for the home confinement condition compared with the reopening condition (*Z* = 12.68, *p* = 0.001) and home confinement condition compared with the semi-lockdown condition (*Z* = 11.77, *p* = 0.001). However, no significant difference was found between the reopening condition and semi-lockdown condition (*Z* = 0.92, *p* = 0.353).

Related to the acceptance of the rules of quarantine and COVID-19 instruction, our findings revealed a significant difference in the COVID-19 condition [χ^2^(2) = 27.20, *p* = 0.001]. Furthermore, *post hoc* comparison by Wilcoxon Signed Ranks tests indicated that this variable was significantly different for the home confinement condition compared with the reopening condition (*Z* = 2.70, *p* = 0.007) and home confinement condition compared with the semi-lockdown condition (*Z* = 5.25, *p* = 0.001). There was a significant difference between the reopening condition and the semi-lockdown (*Z* = 3.09, *p* = 0.001).

The COVID-19 infection factor was not statistically significant in three studied conditions (Cochran’s *Q* = 0.33, *p* = 0.846). In this regard, McNemar tests as for *post hoc* comparison did not show any significant difference between the studied conditions (all *p* > 0.05).

The repeated measure ANOVA showed a significant difference for COVID-19 condition in training during the pandemic (*F*[2,844] = 8.18, *p* = 0.001). Bonferroni correction for multiple comparisons revealed a significant difference between the home confinement condition and the reopening condition (*p* = 0.001), as well as the home confinement condition and the semi-lockdown condition (*p* = 0.047). There was no significant difference between the reopening condition and the semi-lockdown (*p* = 0.308).

The coaches follow-up variable did not show any significant change between the three conditions [χ^2^(2) = 1.64, *p* = 0.438]. In this regard, Wilcoxon Signed Ranks tests as *post hoc* comparison confirmed this finding (home confinement condition vs. reopening condition: *Z* = 0.20, *p* = 0.839; home confinement condition vs. semi-lockdown condition: *Z* = 1.10, *p* = 0.271; reopening condition vs. semi-lockdown: *Z* = 1.34, *p* = 0.180).

The analyses yielded significant differences in need for psychosocial services variable [χ^2^(2) = 17.15, *p* = 0.001]. Subsequently, Wilcoxon Signed Ranks tests as *post hoc* comparison showed that the need for psychosocial services was significantly different for the home confinement condition compared with the semi-lockdown condition (*Z* = 4.41, *p* = 0.001) and the reopening condition compared with the semi-lockdown condition (*Z* = 4.21, *p* = 0.001). However, no significant difference was observed between the home confinement condition and the reopening condition (*Z* = 1.61, *p* = 0.108).

The means and standard deviations for the self-report measures (general health questioners-28, life satisfaction, and mood states) for each of the studied conditions are shown in Table 3.

**TABLE 3:** Changes in factors of General health questionnaire-28, Life satisfaction and Mood states for the three phases of study and results from Friedman test.

**Variable/phase**	**Home confinement condition**		**Reopening condition**		**Semi lockdown condition**		**χ^2^**	***p***
	**(*N* = 525)**		**(*N* = 464)**		**(*N* = 428)**			
	**Mean**	**±*SD***	**Mean**	**±*SD***	**Mean**	**±*SD***		
**General health questioners-28 scores**								
Anxiety and insomnia	9.11	2.00	7.01	2.00	8.85	2.48	153.39	0.001
Somatic symptoms	10.06	2.67	8.54	2.29	9.13	2.86	54.35	0.001
Social impairment	13.33	2.85	10.34	3.32	11.85	2.56	100.52	0.001
Depression	12.59	3.46	9.49	2.28	11.99	2.62	143.10	0.001
Total score	45.10	5.89	35.38	5.13	41.82	5.25	337.72	0.001
**Life satisfaction score**	16.18	4.61	17.34	4.18	18.65	5.48	38.18	0.001
**Mood states scores**								
Confusion	7.08	0.64	6.66	0.47	6.82	0.69	123.92	0.001
Anger	7.58	0.76	7.07	0.75	7.27	0.82	108.40	0.001
Depression	8.66	0.47	7.41	0.49	7.59	0.49	839.98	0.001
Vigor	5.58	0.63	6.67	1.03	5.83	1.03	365.88	0.001
Fatigue	8.33	0.85	7.42	0.63	8.08	0.76	314.48	0.001
Tension	8.16	0.98	7.07	0.86	7.81	0.69	280.08	0.001

As shown in Table 3, Friedman tests showed significant influence of the studied phases on anxiety and insomnia [χ^2^(2) = 153.39, *p* = 0.001], somatic symptoms [χ^2^(2) = 54.35, *p* = 0.001], social impairment [χ^2^(2) = 100.52, *p* = 0.001], depression [χ^2^(2) = 143.10, *p* = 0.001], and total score [χ^2^(2) = 337.72, *p* = 0.001].

**FIGURE 1:**
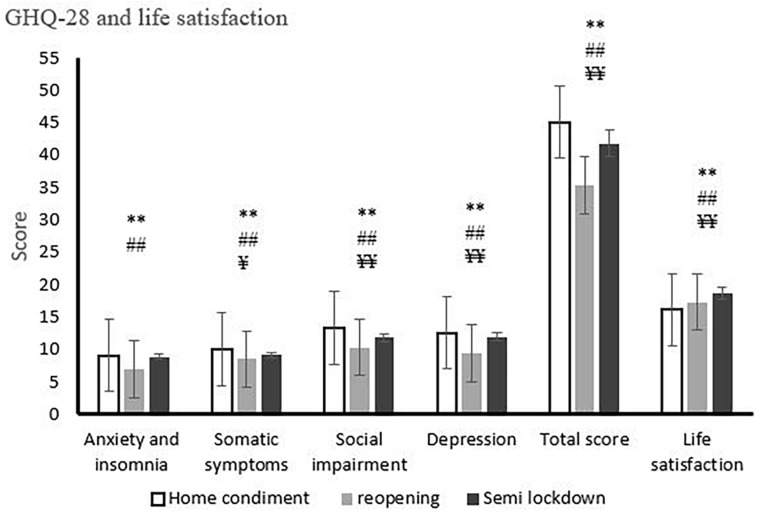
General health questionnaire-28 subscales and life satisfaction during phases of home confinement, reopening, and semi-lockdown conditions. *p < 0.05 (between home confinement condition and reopening condition), **p < 0.01 (between home confinement condition and reopening condition), ^#^*p* < 0.05 (between home confinement condition and semi-lockdown condition), ^##^*p* < 0.01 (between home confinement condition and semi-lockdown condition), ^¥^*p* < 0.05 (between reopening condition and semi-lockdown condition), ^¥¥^*p* < 0.01 (between reopening condition and semi-lockdown condition). Each column represents the mean ratings ± S.E. (standard error).

Figure 1 presents findings from the general health questionnaire-28 subscales and life satisfaction during the phases of home confinement, reopening, and semi-lockdown conditions. *Post hoc* comparison analysis with Wilcoxon Signed Ranks tests revealed that in home confinement condition, GHQ-28 subscales and total score were higher (except for anxiety and insomnia between home confinement condition and semi-lockdown condition: *Z* = 1.27, *p* = 0.203), whereas in the reopening condition (anxiety and insomnia: *Z* = 12.33, *p* = 0.001; somatic symptoms: *Z* = 8.13, *p* = 0.001; social impairment: *Z* = 11.16, *p* = 0.001; depression: *Z* = 11.99, *p* = 0.001; total score: *Z* = 16.30, *p* = 0.001) and in the semi-lockdown condition (somatic symptoms: *Z* = 4.92, *p* = 0.001; social impairment: *Z* = 6.74, *p* = 0.001; depression: *Z* = 2.52, *p* = 0.012; total score: *Z* = 7.67, *p* = 0.001), those parameters were lower. Moreover, there was a significant difference between the reopening condition and the semi-lockdown condition (anxiety and insomnia: *Z* = 10.37, *p* = 0.001; somatic symptoms: *Z* = 3.12, *p* = 0.002; social impairment: *Z* = 6.35, *p* = 0.001; depression: *Z* = 11.73, *p* = 0.001; total score: *Z* = 14.11, *p* = 0.001).

At the home confinement condition, our results using Kruskal–Wallis test indicated that there is no significant difference between age categories [anxiety and insomnia: χ^2^(4) = 2.79, *p* = 0.593; somatic symptoms: χ^2^(4) = 4.42, *p* = 0.328; social impairment: χ^2^(4) = 1.49, *p* = 0.828; depression: χ^2^(4) = 6.38, *p* = 0.172; total score: χ^2^(4) = 3.89, *p* = 0.441] and competitive levels [anxiety and insomnia: χ^2^(2) = 0.323, *p* = 0.851; somatic symptoms: χ^2^(2) = 3.34, *p* = 0.178; social impairment: χ^2^(2) = 1.46, *p* = 0.482; depression: χ^2^(2) = 0.87, *p* = 0.957; total score: χ^2^(2) = 0.56, *p* = 0.756]. According to the Mann–Whitney *U* test, no significant difference was found in gender (anxiety and insomnia: *Z* = 2.01, *p* = 0.053; somatic symptoms: *Z* = −0.37, *p* = 0.710; social impairment: *Z* = −1.11, *p* = 0.264; depression: *Z* = −0.91, *p* = 0.359; total score: *Z* = −0.57, *p* = 0.564) and types of sport (anxiety and insomnia: *Z* = −1.46, *p* = 0.143; somatic symptoms: *Z* = −0.16, *p* = 0.868; social impairment: *Z* = −1.28, *p* = 0.221; depression: *Z* = −0.15, *p* = 0.878; total score: *Z* = −0.1.98, *p* = 0.056).

Under the reopening condition, our results using Kruskal–Wallis test indicated that there is no significant difference between age categories [anxiety and insomnia: χ^2^(4) = 7.74, *p* = 0.101; somatic symptoms: χ^2^(4) = 7.36, *p* = 0.118; social impairment: χ^2^(4) = 1.24, *p* = 0.871; depression: χ^2^(4) = 7.52, *p* = 0.111; total score: χ^2^(4) = 1.08, *p* = 0.896] and competitive levels [anxiety and insomnia: χ^2^(2) = 2.499, *p* = 0.287; somatic symptoms: χ^2^(2) = 1.05, *p* = 0.589; social impairment: χ^2^(2) = 4.48, *p* = 0.106; depression: χ^2^(2) = 0.50, *p* = 0.777; total score: χ^2^(2) = 3.56, *p* = 0.168]. According to the Mann–Whitney *U* test, no significant difference was found in gender (anxiety and insomnia: *Z* = −0.81, *p* = 0.416; somatic symptoms: *Z* = −1.37, *p* = 0.168; social impairment: *Z* = −0.54, *p* = 0.586; depression: *Z* = −1.76, *p* = 0.077; total score: *Z* = −0.76, *p* = 0.442) and types of sport (anxiety and insomnia: *Z* = −0.22, *p* = 0.826; somatic symptoms: *Z* = −0.30, *p* = 0.759; social impairment: *Z* = −1.41, *p* = 0.157; depression: *Z* = −0.63, *p* = 0.524; total score: *Z* = −1.30, *p* = 0.191).

The same results were found for semi-lockdown condition in age categories [anxiety and insomnia: χ^2^(4) = 3.89, *p* = 0.420; somatic symptoms: χ^2^(4) = 5.57, *p* = 0.223; social impairment: χ^2^(4) = 4.54, *p* = 0.337; depression: χ^2^(4) = 4.65, *p* = 0.312; total score: χ^2^(4) = 4.72, *p* = 0.330], competitive levels [anxiety and insomnia: χ^2^(2) = 1.128, *p* = 0.569; somatic symptoms: χ^2^(2) = 2.77, *p* = 0.250; social impairment: χ^2^(2) = 1.53, *p* = 0.465; depression: χ^2^(2) = 2.00, *p* = 0.366; total score: χ^2^(2) = 0.61, *p* = 0.735], gender (anxiety and insomnia: *Z* = −0.82, *p* = 0.410; somatic symptoms: *Z* = −0.50, *p* = 0.614; social impairment: *Z* = −0.69, *p* = 0.486; depression: *Z* = −1.68, *p* = 0.099; total score: *Z* = −1.66, *p* = 0.96), and type of sport (anxiety and insomnia: *Z* = −1.62, *p* = 0.105; somatic symptoms: *Z* = −4.14, *p* = 0.253; social impairment: *Z* = −1.69, *p* = 0.090; depression: *Z* = −0.31, *p* = 0.976; total score: *Z* = −1.01, *p* = 0.144).

Our results for life satisfaction scores indicated a significant difference between the studied phases [χ^2^(2) = 38.18, *p* = 0.001]. Furthermore, Wilcoxon Signed Ranks tests as *post hoc* comparison showed that life satisfaction was significantly different for the home confinement condition compared with the reopening condition (*Z* = 3.56, *p* = 0.001) and the home confinement condition compared with the semi-lockdown condition (*Z* = 6.51, *p* = 0.001). Moreover, a significant difference was found between the reopening condition and the semi-lockdown condition (*Z* = 3.60, *p* = 0.001).

The Kruskal–Wallis test indicated that there is no significant difference between age categories in life satisfaction scores in the home confinement condition [χ^2^(4) = 1.27, *p* = 0.875], reopening condition [χ^2^(4) = 4.32, *p* = 0.363], and semi-lockdown condition [χ^2^(4) = 6.48, *p* = 0.166]. The same results were found for competitive levels [home confinement condition: χ^2^(2) = 4.13, *p* = 0.127; reopening condition: χ^2^(2) = 0.39, *p* = 0.821; semi-lockdown condition: χ^2^(2) = 0.81, *p* = 0.666]. No significant difference (based on Mann–Whitney *U* test) was found by gender (home confinement condition: *Z* = −0.91, *p* = 0.367; reopening condition: *Z* = −0.89, *p* = 0.371; semi-lockdown condition: *Z* = −0.26, *p* = 0794) and sport type (home confinement condition: *Z* = −0.30, *p* = 0.761; reopening condition: *Z* = −0.98, *p* = 0.323; semi-lockdown condition: *Z* = −0.50, *p* = 0.612).

**FIGURE 2:**
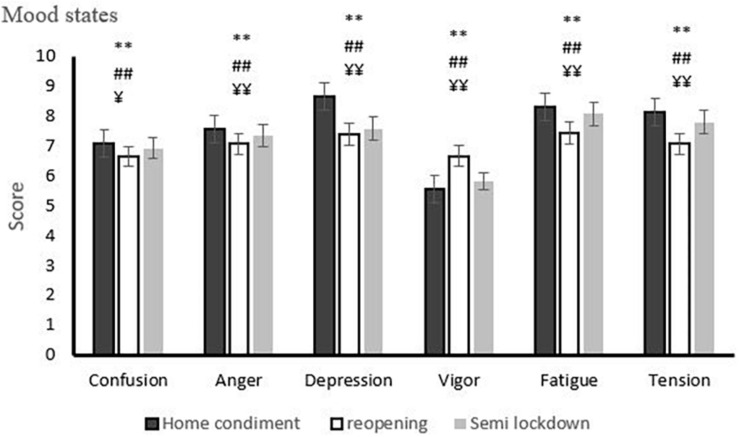
Mood states profile during phases of home confinement, reopening, and semi-lockdown conditions. *p < 0.05 (between home confinement condition and reopening condition), **p < 0.01 (between home confinement condition and reopening condition), ^#^*p* < 0.05 (between home confinement condition and semi-lockdown condition), ^##^*p* < 0.01 (between home confinement condition and semi-lockdown condition), ^¥^*p* < 0.05 (between reopening condition and semi-lockdown condition), ^¥¥^*p* < 0.01 (between reopening condition and semi-lockdown condition). Each column represents the mean ratings ± S.E. (standard error).

The analyses yielded significant differences in confusion [χ^2^(2) = 123.92, *p* = 0.001], anger [χ^2^(2) = 108.40, *p* = 0.001], depression [χ^2^(2) = 839.98, *p* = 0.001], vigor [χ^2^(2) = 365.88, *p* = 0.001], fatigue [χ^2^(2) = 314.48, *p* = 0.001], and tension [χ^2^(2) = 280.08, *p* = 0.001] between the studied conditions. In this regard, Wilcoxon Signed Ranks tests as *post hoc* comparison revealed that mood states scores were significantly different for the home confinement condition compared with the reopening condition (confusion: *Z* = 10.02, *p* = 0.001; anger: *Z* = 20.37, *p* = 0.001; depression: *Z* = 20.30. *p* = 0.001; vigor: *Z* = 15.16, *p* = 0.001; fatigue: *Z* = 14.93, *p* = 0.001; tension: *Z* = 15.08, *p* = 0.001) and for the home confinement condition compared with the semi-lockdown condition (confusion: *Z* = 2.82, *p* = 0.005; anger: *Z* = 11.83, *p* = 0.001; depression: *Z* = 20.21. *p* = 0.001; vigor: *Z* = 4.62, *p* = 0.001; fatigue: *Z* = 6.47, *p* = 0.001; tension: *Z* = 7.66, *p* = 0.001). Moreover, there was a significant difference between the reopening condition compared with the semi-lockdown condition (confusion: *Z* = 6.10, *p* = 0.001; anger: *Z* = 4.08, *p* = 0.001; depression: *Z* = 8.77. *p* = 0.001; vigor: *Z* = 14.38, *p* = 0.001; fatigue: Z = 13.41, *p* = 0.001; tension: *Z* = 13.22, *p* = 0.001) (Figure 2).

At the home confinement condition, our results using Kruskal–Wallis test indicated that there is no significant difference between age categories [confusion: χ^2^(4) = 4.75, *p* = 0.313; anger: χ^2^(4) = 3.47, *p* = 0.482; depression: χ^2^(4) = 5.57, *p* = 0.234; vigor: χ^2^(4) = 3.31, *p* = 0.381; fatigue: χ^2^(4) = 4.18, *p* = 0.381; tension: χ^2^(4) = 1.48, *p* = 0.830] and competitive levels [confusion: χ^2^(2) = 0.52, *p* = 0.768; anger: χ^2^(2) = 0.963, *p* = 0.618; depression: χ^2^(2) = 0.09, *p* = 0.956; vigor: χ^2^(2) = 0.64, *p* = 0.728; fatigue: χ^2^(2) = 0.32, *p* = 0.851; tension: χ^2^(2) = 0.11, *p* = 0.945]. According to the Mann–Whitney *U* test, no significant difference was found by gender (confusion: *Z* = −0.82, *p* = 0.408; anger: *Z* = −1.67, *p* = 0.094; depression: *Z* = −0.96, *p* = 0.337; vigor: *Z* = −0.60, *p* = 0.544; fatigue: *Z* = −0.22, *p* = 0.822; tension: *Z* = −1.16, *p* = 0.130) and types of sport (confusion: *Z* = −0.26, *p* = 0.792; anger: *Z* = −0.14, *p* = 0.989; depression: *Z* = −0.21, *p* = 0.827; vigor: *Z* = −0.56, *p* = 0.572; fatigue: *Z* = −0.57, *p* = 0.569; tension: *Z* = −0.15, *p* = 0.875).

The same results were found for reopening condition in age categories [confusion: χ^2^(4) = 3.80, *p* = 0.433; anger: χ^2^(4) = 2.93, *p* = 0.568; depression: χ^2^(4) = 7.23, *p* = 0.055; vigor: χ^2^(4) = 3.06, *p* = 0.547 fatigue: χ^2^(4) = 2.06, *p* = 0.716; tension: χ^2^(4) = 2.41, *p* = 0.659], competitive levels [confusion: χ^2^(2) = 3.67, *p* = 0.160; anger: χ^2^(2) = 5.286, *p* = 0.071; depression: χ^2^(2) = 5.328, *p* = 0.070; vigor: χ^2^(2) = 4.45, *p* = 0.108; fatigue: χ^2^(2) = 5.14, *p* = 0.076; tension: χ^2^(2) = 3.69, *p* = 0.157], gender [confusion: *Z* = −1.27, *p* = 0.204; anger: *Z* = −0.18, *p* = 0.851; depression: *Z* = −0.23, *p* = 0.816; vigor: *Z* = −0.26, *p* = 0.799; fatigue: *Z* = −1.23, *p* = 0.219; tension: *Z* = −1.76, *p* = 0.078), and types of sport (confusion: *Z* = −0.76, *p* = 0.442; anger: *Z* = −0.44, *p* = 0.660; depression: *Z* = −1.01, *p* = 0.309; vigor: *Z* = −0.35, *p* = 0.724; fatigue: *Z* = −1.40, *p* = 0.159; tension: *Z* = −0.76, *p* = 0.447).

Similar to the previous findings, no significant differences were found in the semi-lockdown condition between age categories [confusion: χ^2^(4) = 4.45, *p* = 0.348; anger: χ^2^(4) = 1.71, *p* = 0.789; depression: χ^2^(4) = 3.06, *p* = 0.546; vigor: χ^2^(4) = 1.57, *p* = 0.813; fatigue: χ^2^(4) = 3.58, *p* = 0.465; tension: χ^2^(4) = 2.73, *p* = 0.603], competitive levels [confusion: χ^2^(2) = 1.72, *p* = 0.423; anger: χ^2^(2) = 1.76, *p* = 0.414; depression: χ^2^(2) = 2.017, *p* = 0.365; vigor: χ^2^(2) = 2.47, *p* = 0.290; fatigue: χ^2^(2) = 0.76, *p* = 0.682; tension: χ^2^(2) = 0.26, *p* = 0.874], gender (confusion: *Z* = −1.42, *p* = 0.153; anger: *Z* = −0.62, *p* = 0.530; depression: *Z* = −1.02, *p* = 0.314; vigor: *Z* = −1.30, *p* = 0.221; fatigue: *Z* = −0.20, *p* = 0.838; tension: *Z* = −1.10, *p* = 0.302), and types of sport (confusion: *Z* = −0.50, *p* = 0.960; anger: *Z* = −0.82, *p* = 0.408; depression: *Z* = −1.07, *p* = 0.282; vigor: *Z* = −0.81, *p* = 0.417; fatigue: *Z* = −0.93, *p* = 0.350; tension: *Z* = −1.66, *p* = 0.095).

Finally, the Δ changes between three phases of the COVID-19 pandemic were calculated from the general health questioners-28, life satisfaction score, and mood states profile. Table 4 shows the correlation between the changes of the assessed variables.

**TABLE 4:** Correlation among mental health, mood states and life satisfaction.

**Variable Δ**	**1**	**2**	**3**	**4**	**5**	**6**	**7**	**8**	**9**	**10**	**11**	**12**
(1) Total score-GHQ	–	0.374**	0.611**	0.475**	0.630**	0.008	−0.014	−0.046	−0.012	−0.108*	−0.035	−0.050
(2) Anxiety and insomnia		–	−0.024	0.071	−0.018	−0.095	−0.029	−0.072	0.015	−0.027	−0.049	0.051
(3) Social impairment			–	0.025	0.178**	0.052	−0.071	−0.039	−0.039	−0.120*	−0.056	−0.029
(4) Somatic symptoms				–	−0.004	0.006	0.029	−0.007	−0.072	0.047	0.012	−0.057
(5) Depression					–	0.024	0.036	0.009	0.062	0.069	0.015	−0.058
(6) Life satisfaction						–	−0.048	−0.0012	−0.001	−0.019	−0.037	0.035
(7) Confusion							–	−0.074	0.556**	−0.062	0.512**	0.482**
(8) Anger								–	0.422**	0.035	0.071	0.386**
(9) Depression									–	0.109*	0.270**	0.255**
(10) Vigor										–	−0.314**	−0.238**
(11) Fatigue											–	0.130**
(12) Tension												–

**p < 0.01, *p < 0.05.

Pearson correlation showed significant positive correlations between total score of GHQ-28 with anxiety and insomnia (*r* = 0.374, *p* = 0.001), social impairment (*r* = 0.611, *p* = 0.001), somatic symptoms (*r* = 0.475, *p* = 0.001), depression (*r* = 0.630, *p* = 0.001), and a negative correlation with vigor (*r* = −0.108, *p* = 0.040). Moreover, there was a positive significant correlation between social impairment with depression (*r* = 0.178, *p* = 0.001) and a negative correlation with vigor (*r* = −0.120, *p* = 0.023). In addition, confusion (*r* = 0.556, *p* = 0.001) and anger (*r* = 0.422, *p* = 0.001) showed significant correlations with depression in mood state profile. There was a significant correlation between anger and tension (*r* = 0.386, *p* = 0.001). Additionally, there were significant correlations between confusion with fatigue (*r* = 0.512, *p* = 0.001) and tension (*r* = 0.482, *p* = 0.001), as well as depression with vigor (*r* = 0.109, *p* = 0.023), fatigue (*r* = 0.270, *p* = 0.001), and tension (*r* = 0.255, *p* = 0.001). The vigor subscales were negatively correlated with fatigue (*r* = −0.314, *p* = 0.001) and tension (*r* = −0.238, *p* = 0.001). Finally, the correlation between fatigue and tension was also found significant (*r* = 0.130, *p* = 0.001). No significant correlation, however, was found between the other tested variables.

## Discussion

The purpose of this study was to analyze the status of mental health, life satisfaction, and mood states of elite athletes affected by the COVID-19 pandemic during the phases of home confinement, reopening, and semi-lockdown conditions. The results illustrated that the rate of COVID-19 infection was not different and the rate was low during the studied phases. It is speculated that a large amount of information is given about COVID-19 and slow adaptation to the pandemic conditions together with the application of the health guidelines and the abundance of health equipment (e.g., facemask and antiseptics) have played a role in controlling the COVID-19 infection rate in the studied population. More research is needed to compare the rate of infection with the general population (and in other sport disciplines) to investigate if the rate is different and what are potential reasons under sports and competition settings.

The acceptance of the quarantine rules and massive instructions related to the pandemic were in general higher in the phases of reopening and semi-lockdown compared with the confinement phase. A case study indicated that sports persons have a positive approach toward this crisis, rules, and instructions in comparison with the rest of the population during the COVID-19 pandemic (Jaenes et al., 2020a; Pandey, 2020). It is noteworthy that most likely for the post-pandemic, there will be many instructions for returning to sports safely and that athletes must follow some guidelines for training (DiFiori et al., 2020; Toresdahl and Asif, 2020). Further studies should be considered to determine the underlying mechanism(s) of acceptance of the rules of COVID-19, in particular in athletes.

As expected, our results indicated that the economic conditions have deteriorated during the COVID-19 pandemic. In more detail, the number of people who have suffered from the economic damage caused by the COVID-19 has increased during the studied phases (e.g., 10.3% in the confinement condition, 52.3% in the reopening condition, and 56.3% in the semi-lockdown conditions). Recent publications have reported similar findings relevant to the economic influence of the COVID-19 pandemic (García-Tascón et al., 2020; Nicola et al., 2020; Polyakova et al., 2020). In the context of elite athletes, Drewes et al. (2020) reported that in those who have sport-based income, 65% indicated that their funding, sponsorship, or salary had been affected. The way in which the athletes had been affected, however, varied. In their study, 44% no longer received any compensation for matches or competitions due to the cancelation of most competitive sports activities. However, there is no similar longitudinal study in the literature that we could compare our data during the COVID-19 pandemic. Researchers have proposed that such economic hardships lead to a much higher prevalence of expressing mental health issues, including feelings of depression and anxiety (Witteveen and Velthorst, 2020). Therefore, governments and sports organizations must consider financial support for the affected athletes and sports teams, not only under the current situation but also for future prevention strategies (Mehrsafar et al., 2020).

The training rate was found to be negatively affected in our study. Despite recommendations that home confinement should not hinder people from being physically active, present results showed a decline in training rate during the COVID-19 home confinement phase. Moreover, we found that the training rate during the reopening phase was higher as compared with the home confinement and semi-lockdown conditions. This finding is in line with previous studies (Pillay et al., 2020). It has been reported that the home confinement period by COVID-19 caused reductions in training volume and intensity and decreased sleep quality in handball players and professional and non-professional football players (Mon-López et al., 2020a,b). Moreover, a multicenter study reported that COVID-19 home confinement had a negative effect on the level of sports activities (Ammar et al., 2020a).

Despite the physical activity guidance provided to athletes (World Health Organization, 2020d), our results indicated that it has not been possible for individuals to adequately maintain their normal sport activity patterns at home or in private clubs. The decline in sport activities was accompanied by increased sedentary behavior (Wong et al., 2020). However, the extent to which training and sports activities are impacted by the current COVID-19 pandemic will be linked to the stringency of individual government confinement policies. However, after the reopening and in the semi-lockdown conditions, the amount of sports activity and training showed an increase, but the level is still very low compared to the pre-pandemic time. One of the most important factors is related to the returning to competition and regular form of sports activities. It has been shown that low tolerance during COVID-19 pandemic conditions caused high levels of dysfunctional response (e.g., anxiety, stress, depression, and alexithymia) in Spanish elite athletes (Hernández et al., 2021). Although reopening and semi-lockdown conditions increase sports activity rates, the fear of infection and restrictions in many sports (e.g., contact sports) are no different from home quarantine. This result is in line with previous research done in New Zealand, which reports that the rate of physical activity was lower at post-lockdown compared to pre-lockdown in highly active people (Hargreaves et al., 2021). Sports at the elite levels require sports equipment, spaces, and the presence of coaches and training opponents. These needs are difficult to meet during the COVID-19 pandemic; it can be one of the reasons for the decline in sports activity in these conditions (Herrero-Gonzalez et al., 2020; Latella and Haff, 2020). Sports organizations and sports medicine professionals need to find solutions for these issues in training and competitions related to elite athletes, especially in sports where competitions and training have not started, such as some sports in which the return to competition has begun. Failure to return to competitive conditions can cause many mental health problems in elite athletes, such as reduced fitness and income (Ernstsen and Havnen, 2020; Mehrsafar et al., 2020). Future sports activities can benefit from the solutions offered by Information and Communications Technology (ICT), such as home-based training and fitness apps (Ammar et al., 2020b). For instance, a study showed that participants who were users of eHealth for exercise and physical activity presented significantly higher levels of vigorous physical activity and total physical activity per week than non-users during the COVID-19 pandemic (Marchant et al., 2021).

Regarding coach–athletes interaction, our elite athletes reported a moderate rate of follow-up and monitoring of the training during the COVID-19 studied phases. A recent study reports that the coaches have experienced a wide spectrum of emotional and cognitive reactions such as disappointment, frustration, confusion, and relief after the postponement of the Tokyo 2020 Olympic and Paralympic Games by the COVID-19 pandemic (Taku and Arai, 2020). Moreover, a group of researchers illustrated that 80% of Italian athletes stayed in contact via the web with their coaches or other professionals during the Italian lockdown caused by the COVID-19 (di Fronso et al., 2020). The coach follow-up can help athletes to feel less stress, avoid behavioral and motivational problems, and reduce negative emotional states. To our knowledge, our study is the first to report coach follow-up in athletes during the COVID-19 pandemic in the three phases of home confinement, reopening, and semi-lockdown. Future studies could investigate whether these findings would be similar in other athletes, and if any difference exists, what can be potentially a reason behind that. Moreover, artificial intelligence and digital-based platforms can potentially provide help and plans for athlete–coach communication and training, and consequently, recommendations for physical and mental fitness and return to sports competition can be provided (Lim, 2020).

As recently have been discussed by Van Bavel et al. (2020), the widespread term “social distancing,” implying that one needs to cut off meaningful interactions, should be fully replaced by “physical distancing.”. Additionally, some athletes have stated that they are isolated and feel lonely, which has impacted their mental health (Gorczynski and Aron, 2020). In line with these challenges, our result highlighted that the elite athletes have been in constant need of psychosocial services throughout the COVID-19 pandemic. A narrative review by Reardon et al. (2020) has also noted that the COVID-19 pandemic has created changes in dealing with mental health symptoms and disorders in elite athletes, as a special population, to receive specific psychosocial services. This review has recommended that within the realm of psychotherapy, crisis counseling, and other forms of individual psychotherapy, couple/family and group psychotherapy might be helpful during the COVID-19 pandemic (Reardon et al., 2020). Besides, Psychological First Aid (PFA), telemental health, and video conference, plus other virtual health care interventions can be utilized in line with the psycho-social approach (Reardon et al., 2020; Yang et al., 2020). Furthermore, media co-creation, following the advice shared by athletes and teams, engagement with challenges, and fundraising contributions can be a new direction in social services, social responsibility, and altruistic behavior (Sharpe et al., 2020). In particular, messages on social media promoting hygiene, physical distancing, and sport provide a simple yet productive way for athletes and sports organizations to connect with sports fans and contribute to the global effort to slow down the spread of COVID-19.

The WHO has warned communities about the pandemic-related mental health problems among the general public (World Health Organization, 2020c). Studies in the field of sports have also emphasized the importance of this issue in elite athletes (Mehrsafar et al., 2020; Reardon et al., 2020). The result of our study indicated that home confinement caused a negative impact on the mental health status of elite athletes. In more detail, we found that the anxiety and insomnia, somatic symptoms, social impairment, and depression were the highest negatively affected parameters during the home confinement phase as compared with the reopening and semi-lockdown phases. This finding is in line with the previous studies that have reported a decreased level of mental health parameters in athletes (Bowes et al., 2020; Håkansson et al., 2020; Lamberts and Gomez-Ezeiza, 2020; Makarowski et al., 2020; Pillay et al., 2020). A study has reported that the martial art athletes did not engage frequently in active coping strategies, such as planning, or positive reframing during the COVID-19 pandemic (Makarowski et al., 2020). This approach may put elite athletes in a negative cycle that puts mental health at risk. Moreover, acceptance of the COVID-19 situation might be linked to stress-related growth among the athletes; in this situation, athletes think that they are in a dark period of their professional life and it is difficult for them to return to previous fitness and competitive level, which can be a source of stress and anxiety. It has been known that it may cause anxiety and post-traumatic stress symptoms in athletes similar to non-athletes (Şenışık et al., 2020). Besides, spending a long time at home during home confinement and reduction of communication have reduced the level of social support for elite athletes in general. These observations and reports point to a reduced mental health situation with a potential increased psychological disorder symptom known as “adding stress to the stressed.” Since physical activity is recognized with antidepressant and anxiolytic effects, this change alone could substantially worsen the elite athletes’ mental health. Sports activity has been considered a way in preventing psychiatric symptoms and poor mental health (Reardon et al., 2020). Therefore, sports organizations, coaches, and other guideline providers should consider proper plans for elite athletes to optimize their training programs. Moreover, a safe strategy with risk–benefit evaluation on how to return to competitions would help restore both mental and physical health in this population. On the other hand, planning for monitoring, evaluation, intervention, and management of mental health should be considered with a high priority. In this vein, interventions can benefit from a variety of psychotherapy approaches to psychiatric therapies with a specific approach to fit the elite athletes’ needs (Reardon et al., 2020).

Our study revealed that life satisfaction changed to the home confinement, reopening, and semi-lockdown phases. Life satisfaction after the home confinement period showed an increase over time, where the highest rate was at the semi-lockdown condition. A multicenter study also reports a negative impact of home confinement on the life satisfaction scores (Ammar et al., 2020a) and the fact that concerns about the illness and COVID-19 infection can be associated with lower life satisfaction (Von Soest et al., 2020). Additionally, physically active people might be more susceptible to well-being issues and lower life satisfaction during a lockdown (Zhang and Tower, 2020). However, one must also consider that we studied the home confinement phase along with the reopening and semi-lockdown conditions, and there is no similar study in the literature that we could compare our data. Different studied populations under different conditions posed by the COVID-19 pandemic and different instruments to study the influence of the pandemic on mental health in athletes make it difficult for comparisons between studies at this time point. However, a general trend of the negative effects of the pandemic on mental health seems currently accepted.

Some speculations can help to explain these results. Feeling dissatisfied with life during the home quarantine period might be a result of the social distancing from emotionally attached individuals, such as team members and coaches, and the inability to engage in professional sports activities, which is known as psychological stress that can eventually lead to dissatisfaction with life. In addition, adapting to the COVID-19 conditions and improving the mood and, to some extent, returning to continued activities and sports (albeit with limitations) can probably be among other reasons to improve life satisfaction. Therefore, to maintain an acceptable level of life satisfaction, it is important to stay in touch and be able to pursue activities during the COVID-19 condition while physical distance is maintained. In this regard, a longitudinal study indicated that athletes have a tendency toward a psychological adaptation to the stressful conditions they have to face (Rubio et al., 2020). Providing online meetings to reduce or close the gap can be helpful. More research is needed to find strategies to improve the quality of life during the COVID-19 pandemic; however, the puzzle seems complex with multiple layers of confounding factors.

The effect of COVID-19 on the mood responses in elite athletes is an important indicator of how athletes are coping with the pandemic. We found that the pattern of mood responses reflected as an inverse iceberg profile, characterized by significantly elevated scores for depression, anger, fatigue, tension, and confusion, and lower scores for vigor during all studied phases. However, the vigor subscale was higher in the reopening condition, whereas the depression, anger, fatigue, tension, and confusion subscales were lower. Moreover, depression, anger, fatigue, tension, and confusion subscales during the home confinement revealed the highest scores compared with the other phases of the study. According to the inverse iceberg profiles that have been reported in the literature to increase the risk of psychopathology (Terry et al., 2020) or cause failure and reduce well-being in elite athletes (Terry and Parsons-Smith, 2019), we assume that these can also explain what we observed in this study. A recent survey indicated that tension, depression, anger, fatigue, confusion, and reduced vigor were identified in 1,062 Australian people during the periods of COVID-19 restrictions, representing significant mood disturbances (Terry et al., 2020). Moreover, Mon-López et al. (2020b) showed that home confinement affects training load, the recovery process, and mood states in top-level football players.

There are several plausible explanations for the observed increase in negative feeling states. The pandemic has undoubtedly caused fear and loss for many elite athletes; health fears for self and loved ones, fear of isolation, loss of income, or social support, and a sense of lack of normality (Mehrsafar et al., 2020; Terry et al., 2020). During this pandemic, elite athletes have lost livelihoods, relationships, and opportunities for participation in national and international tournaments, or have been denied access to simple activities that give them pleasure, such as physical contact with friends and family, or interacting with sports colleagues. In this regard, a reluctance or inability to share grief and loss with others may be associated with mood decrements and increased potential for psychopathology to open a window of vulnerability (Terry et al., 2020). Moreover, the anti-negative mood effect of exercise training has a strong evidence base (Siqueira et al., 2016; Jaenes et al., 2020b). In this line, reduced exercise duration during the pandemic has been associated with higher scores in depression, anxiety, and stress as well as negative mood states (Mon-López et al., 2020b; Stanton et al., 2020). Therefore, physical activity (and even virtual reality exercise) could be recommended to reduce the negative emotional effect in periods of confinement and COVID-19-related restrictions (Gao et al., 2020). In addition, adaptive strategies such as acceptance, reappraisal, and problem-solving in a different setting (e.g., ICT and digital interventions) are techniques increasingly being used under the current condition (Aldao et al., 2010).

### Strengths and Limitations

Previous studies have suggested that studies in the field of COVID-19 in elite athletes must be performed with a follow-up setting. We assessed the elite athletes during the home confinement, reopening, and semi-lockdown phases, which allowed us to analyze the dynamic changes that occurred overtime. This expanded our understanding of the needs and challenges of this special population and could show a diversity of the negative impact on elite athletes during the three studied phases.

The present study has limitations, mainly associated with the fact that this is an anonymous online survey, with limited possibilities for in-depth questions or full diagnostic instrument to be used, and a relatively low response rate as well as truthful answers. Moreover, we utilized online data collection based on social media platforms and internet pages. Several studies have recommended that an online survey research can be helpful under crisis more than a face-to-face method, and this method has some advantages during COVID-19 conditions (e.g., prevent transmission of infection, travel, and quarantine restrictions, time, cost, large sample size, and repetition of study) (Wright, 2005; Rice et al., 2017). However, there are several disadvantages that are mentioned in previous studies (e.g., this method is not able to guarantee the pure sampling and accurate demographic or characteristic information) (Rice et al., 2017). For solving this limitation, future studies can get the membership lists of athletes from sports organizations and send an invitation link to recognized athletes. Additionally, next studies should consider more rigorous methods for data collection (e.g., personal surveys, telephone surveys, and mobile software surveys). It should also be noted that our questioners are not replacements of diagnostic tools, and hence, although our results may signal an increased risk of clinical psychopathology among elite athletes, they could equally be seen in society. Accordingly, comparing the results with the general population can better determine the pandemic status of COVID-19 in elite athletes. Besides, our study used only questionnaires, other studies can use physiological markers (e.g., immunity and hormonal markers) that are related to mental health. In this regard, studies with a mixed methodology can be used in the future for better diagnosis.

We did not include a status of the pre-lockdown condition about mental health, life satisfaction, and mood state profiles; thus, our present findings cannot be compared to those parameters of the pre-lockdown condition.

Future study designs could consider including more variables about returning to the competition [duration and intensity of training, monitoring systems for the training quantity and quality, competitive camps, place (home or away competition), COVID-19 infection during the competitive phase, isolation of elite athletes by COVID-19 infection, etc.] and new restrictions posed during this pandemic. Besides, comparing results in different countries with different languages and cultures, per capita income levels, diverse support, and diverse constraints over a single period or at different times can help to integrate conclusions. Future studies can also be conducted experimentally and a variety of psycho-behavioral interventions (under an ICT setting or face-to-face and even group therapy) and training plans for elite athletes can be applied to investigate if any improvement would show up for mental health factors and physical fitness.

## Conclusion

Findings from this explorative study suggest that mental health, life satisfaction, and mood states were influenced during the different phases of the COVID-19 pandemic. Reopening and semi-lockdown phases were found to be associated with higher mental health, mood state, and life satisfaction. Home confinement created problems with trainings and plans and economic damage. Overall, this study provided the first evidence on dynamic changes of the COVID-19 phases on several psychological factors in elite athletes. According to the results, specific programs to support elite athletes at a psychological level could be recommended, especially to improve training rate, mental health, mood states, and life satisfaction. Accordingly, safe conditions and a rigorous monitoring system must be established by federations and institutions to protect the integrity of elite athletes. Learning from the past crises caused by the previous pandemics, planning for the current situation, and eventual future strategies seem crucial.

## Data Availability Statement

The raw data supporting the conclusions of this article will be made available by the authors, without undue reservation.

## Ethics Statement

The studies involving human participants were reviewed and approved by the Ethics Committee of the Tehran University of Medical Sciences (number: 1399-335) and was conducted according to the Declaration of Helsinki. The patients/participants provided their written informed consent to participate in this study.

## Author Contributions

JCJS conceived the original study in Spain. AM, AZ, PG, MN, and MT conceived the study and collected the data. AM, MT, MA, and AZ designed the questionnaire and analyzed the data. All authors wrote the manuscript. AM, AZ, PG, JCJS, MN, and MA provided critical revisions on the successive drafts. All authors read and approved the final manuscript.

## Conflict of Interest

The authors declare that the research was conducted in the absence of any commercial or financial relationships that could be construed as a potential conflict of interest.
